# Angiogenesis revisited from a metabolic perspective: role and therapeutic implications of endothelial cell metabolism

**DOI:** 10.1098/rsob.170219

**Published:** 2017-12-20

**Authors:** Nihed Draoui, Pauline de Zeeuw, Peter Carmeliet

**Affiliations:** 1 Laboratory of Angiogenesis and Vascular Metabolism, Department of Oncology, KU Leuven, Leuven 3000, Belgium; 2 Laboratory of Angiogenesis and Vascular Metabolism, Center for Cancer Biology, VIB, KU Leuven, Campus Gasthuisberg O&N4, Herestraat 49-912, Leuven 3000, Belgium

**Keywords:** endothelial cell metabolism, angiogenesis, PFKFB3, CPT1a, GLS1, ASNS

## Abstract

Endothelial cell (EC) metabolism has lately emerged as a novel and promising therapeutic target to block vascular dysregulation associated with diseases like cancer and blinding eye disease. Glycolysis, fatty acid oxidation (FAO) and, more recently, glutamine/asparagine metabolism emerged as key regulators of EC metabolism, able to impact angiogenesis in health and disease. ECs are highly glycolytic as they require ATP and biomass for vessel sprouting. Notably, a regulator of the glycolytic pathway, 6-phosphofructo-2-kinase/fructose-2,6-bisphosphatase 3, controls vessel sprouting during the angiogenic switch and its inhibition in tumour ECs leads to vessel normalization, thereby reducing metastasis and ameliorating chemotherapy. Moreover, FAO promotes EC proliferation through DNA synthesis, and plays an essential role in lymphangiogenesis via epigenetic regulation of histone acetylation. Pathological angiogenesis was decreased upon blockade of carnitine palmitoyltransferase 1, a regulator of FAO in ECs. More recently, metabolism of glutamine, in conjunction with asparagine, was reported to maintain EC sprouting through TCA anaplerosis, redox homeostasis, mTOR activation and endoplasmic stress control. Inactivation or blockade of glutaminase 1, which hydrolyses glutamine into ammonia and glutamate, impairs angiogenesis in health and disease, while silencing of asparagine synthetase reduces vessel sprouting *in vitro*. In this review, we summarize recent insights into EC metabolism and discuss therapeutic implications of targeting EC metabolism.

## 1. The endothelium: a truly remarkable organ

Diffusion is an energetically inexpensive means for oxygen and nutrient supply as well as for waste disposal, but is slow and limited in spatial distance [1]. Therefore, evolutionary increases in organism size have mandated the formation of a circulatory system to conduit moving fluids and gasses to and from distant tissues. A blood vascular circulatory system is believed to have first appeared over 600 million years ago. Initially, blood vessels in invertebrates were simply matrix channels without an endothelial lining, and therefore carried only slow turbulent blood flow, sufficient, however, to meet their low metabolic demands [1]. Approximately 100 million years later, some types of invertebrates evolved to more rapidly moving predators, requiring higher metabolic needs. To meet these increased metabolic demands, blood vessels became lined with a smooth endothelial layer, thereby creating the endothelium, now permitting laminar and therefore also more rapid blood flow—and most importantly establishing continuous supply and delivery of nutrients and oxygen to all tissues of organisms for more efficient metabolic production of energy and biomass [1]. As such, the vascular circulatory system (comprising blood and lymphatic vessels) in a human adult has evolved into a closed and intricate network of arteries, veins and capillaries which, when tied together, add up to a truly remarkable length of 90 000 km, the equivalent of circling the Earth 2¼ times [2,3]. This makes the endothelium one of the largest organs in the human body. In addition, tissues rely on blood vessels for pH and temperature homeostasis, while lymphatic vessels mainly control the absorption and filtering of interstitial fluids and mediate immune surveillance [2,3]. Clearly, the era of perceiving the endothelium as a mere selectively permeable interface between blood and its surrounding tissues has come to an end.

Interestingly, the vasculature is one of the primary functional organs to be formed during early embryogenesis through a complex but strictly coordinated process called vasculogenesis [4]. Once a primitive vascular plexus is formed via vasculogenesis (de novo vessel formation derived from mesodermal angioblasts), neovessel formation can take place from the existing primary vascular plexus via two mechanisms: (i) intussusception, the process of vessel splitting; or (ii) angiogenesis, the process of vessel sprouting [4,5]. The latter process is critical during not only early embryogenesis and further development, but also healthy adulthood for the female monthly reproductive cycle, for placental growth during pregnancy and for wound healing [6]. Endothelial cells (ECs) are the building blocks of vessels and as such key are players in sprouting angiogenesis. As the endothelium plays a fundamental role in maintaining tissue homeostasis, EC dysfunction contributes to a panoply of diseases—more than any other organ in our body upon its dysfunction [7,8]. For instance, excessive angiogenesis promotes pathologies including cancer, pulmonary arterial hypertension (PAH), inflammatory disorders and ocular diseases; while insufficient vessel sprouting results in ischaemia, promoting pathologies such as myocardial infarction, stroke and neurodegenerative or obesity-associated disorders (diabetic and atherosclerotic vasculopathies).

## 2. Angiogenesis and its therapeutic potential

In adult humans, healthy established vessels are lined by a single monolayer of quiescent ECs, able to retain this state of quiescence for years. However, upon ischaemia (nutrient deprivation and hypoxia) or tissue injury (inflammation), quiescent ECs are able to rapidly switch into a proliferative/angiogenic state in order to restore proper nutrient and oxygen delivery necessary for tissue homeostasis and to supply immune cells required for wound healing [6]. This process of angiogenesis relies on a highly coordinated orchestra of regulatory mechanisms, accomplished mainly by three distinct EC subtypes. Briefly, a pro-angiogenic stimulus (i.e. vascular endothelial growth factor (VEGF), fibroblast growth factor (FGF)) activates the nearest EC type—the ‘tip cell’—from an existing vessel, which guides the newly forming vessel sprout towards the source of the pro-angiogenic stimulus via its migratory (non-proliferative) phenotype and numerous filopodia and lamellipodia. While migrating, the tip cell's multiple neighbouring ‘stalk cells’ elongate the neovessel sprout through their proliferative (non-migratory) phenotype. Once neighbouring neovessel sprouts meet and their tip cells fuse (through a process called anastomosis, regulated in part by VEGF-C), an interconnected lumen and subsequent closed system is formed, allowing functional blood flow [9]. Finally, the quiescent ‘phalanx cells’ define the mature part of the neovessel characterized by a typical cobblestone shape [5]. Such quiescence aiming to form a tight monolayer for proper stability and barrier function is further established by the secretion of platelet-derived growth factor (PDGF)-B and subsequent recruitment of PDGF receptor β expressing vascular smooth muscle cells, such as pericytes [10].

Remarkably, this tip versus stalk cell specification is highly dynamic as the fittest EC always takes on the tip cell position leading the neovessel sprout at any given moment—in a process called ‘tip cell overtaking’, primarily controlled by a VEGF–Delta-like ligand 4 (Dll4)–Notch signalling cascade [11,12]. Among all pro-angiogenic stimuli, VEGF is the key regulator of vascular function and EC subtype specification. Briefly, once the continuous VEGF gradient reaches the nearest vessel, VEGF binds its receptor VEGFR2 on the nearest EC, destining it to become a tip cell. Upon VEGF–VEGFR2 binding, the expression of the Notch receptor ligand Dll4 is induced in the tip cell. Tip cell-derived Dll4 binds to its Notch receptor presented on the neighbouring ECs, which adopt a stalk cell phenotype. Dll4–Notch binding in these stalk cells induces the cleavage and release of the Notch intracellular domain (NICD), which consequently downregulates the expression of VEGF receptors VEGFR2 and VEGFR3, and co-receptor neuropilin-1, while upregulating VEGFR1 expression—a transcriptional outcome resulting in reduced VEGF responsiveness and enforcing the stalk cell phenotype [13]. As previously mentioned, this EC specification is a highly dynamic process in which the tip cell is the EC type with the highest VEGF sensitivity (i.e. highest VEGFR2/VEGFR1 expression ratio). Considering the pivotal role played by VEGF in angiogenesis and EC subtype specification, extensive research has primarily been focused on cellular and molecular mechanisms underlying VEGF-regulated angiogenesis in health and disease [14–16]. Several anti-angiogenic strategies targeting VEGF have been clinically approved for the treatment of various types of cancer and ocular diseases [17]. Unfortunately, the success of such anti-angiogenic therapies is limited by insufficient efficacy and resistance.

Although traditionally believed to be governed solely by genetic signalling cascades (i.e. VEGF–Dll4–Notch), the switch from a quiescent to a proliferative/angiogenic state is accompanied by a ‘metabolic switch’ in ECs. It was only recently that EC metabolism arose as a new essential regulator of vascular function and EC behaviour [18–24]. Recent scientific investigations deciphered that blood/lymphatic vessel sprouting mechanisms and physiological/pathological angiogenic processes rely on the enhancement of particular endothelial metabolic pathways involving glucose, fatty acids (FAs) and glutamine (the latter also linked to asparagine metabolism) [18–24]. Essentially, such metabolic adaptation makes sense considering each EC subtype's different energetic needs. These novel findings illustrated the critical role of endothelial metabolic enzymes including 6-phosphofructo-2-kinase/fructose-2,6-bisphophatase (PFKFB3), carnitine palmitoyltransferase 1a (CPT1a), glutaminase 1 (GLS1) and asparagine synthetase (ASNS) in angiogenesis, as well as the regulatory role of the transcription factor FOXO1 ensuring EC quiescence and vascular homeostasis [25]. The following sections provide a comprehensive summary of the latest studies on EC metabolism, including its therapeutic opportunities.

## 3. Metabolic pathways and new therapeutic targets in ECs

### 3.1. Glycolysis and PFKFB3

**Figure 1.**
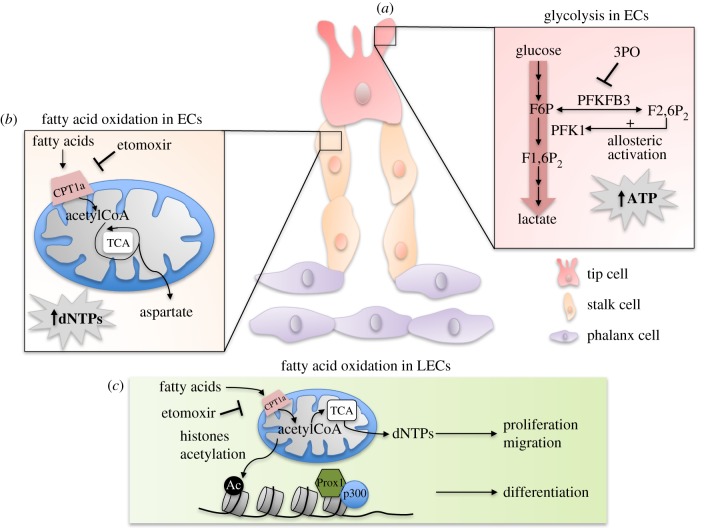
PFKFB3-driven glycolysis and the central role of FAO via CPT1a function in ECs (*a*) Schematic of PFKFB3 as a key regulator of the glycolytic activity in ECs and the angiogenic switch. F2,6P_2_, produced from F6P by PFKFB3, allosterically activates PFK1 in order to further increase glycolytic flux and quickly generate high amounts of ATP necessary for ECs to sprout. (*b*) Illustration of FAO metabolic pathway through the rate-controlling enzyme CPT1a in vessel sprouting. FAO and CPT1a are necessary during vessel sprout elongation to replenish the TCA cycle and to produce aspartate as a precursor of dNTPs for cell replication. (*c*) FAO and CPT1a are pivotal for lymphangiogenesis by promoting DNA synthesis, and thus proliferation, but also for venous-to-lymphatic EC differentiation through histone acetylation and epigenetic mechanisms.

Angiogenesis is an energy- and biomass-demanding process. Under physiological conditions of quiescence, ECs already have high glycolytic activity (as a primary source of ATP) [18]. However, under angiogenic conditions, proliferating and migrating ECs upregulate their glycolytic activity even further as migratory tip cells require high levels of ATP to remodel the actin cytoskeleton in its filopodia and lamellipodia [18] (figure 1*a*). Glycolysis metabolizes glucose into two pyruvate molecules (exported from the cell as lactate) and has a net generation of 2 mol of ATP. In ECs, glycolytic flux was estimated to be ±200-fold higher than glucose oxidation flux, despite glucose oxidation's (via oxidative phosphorylation (OXPHOS) in mitochondria) 16–18-fold higher net ATP yield per mole of glucose [18,26]. In addition, ECs die when deprived of glucose, thus illustrating that they are glycolysis-addicted.

Selecting glycolysis over OXPHOS, however, may hold advantages: (i) high glycolytic flux results in high lactate concentrations, a metabolite used for pro-angiogenic signalling; (ii) high glycolytic flux can yield higher amounts of ATP in a shorter time frame when glucose levels are not limiting—this is especially valuable when new sprouts need to quickly re-vascularize ischaemic tissues; (iii) filopodia and lamellipodia are thin structures requiring high levels of ATP for cytoskeleton remodelling, however they lack space to fit the relatively large mitochondria; (iv) metabolism via OXPHOS would increase noxious reactive oxygen species (ROS); and (v) metabolism via glycolysis rather than OXPHOS preserves maximal oxygen levels for transfer to surrounding perivascular cells [7,27].

During angiogenesis, VEGF stimulation results in the elevation of glycolytic flux and displays increased expression of glucose transporter 1 (GLUT-1) and of the glycolytic enzymes lactate dehydrogenase A (LDH-A) and notably PFKFB3 [18,28,29]. PFKFB3 has been designated a key glycolytic regulator in ECs. Although it is not directly part of the glycolytic pathway, its kinase activity (700-fold higher than its phosphatase activity) generates fructose-2,6-bisphosphate (F2,6BP), thereby allosterically activating phosphofructokinase 1 (PFK-1), a direct rate-limiting glycolytic enzyme. The necessity of glycolytic flux in angiogenic ECs (in both tip and stalk cells) is underlined by the impaired *in vitro* EC spheroid sprouting in combination with *in vivo* postnatal outgrowth and branching of murine retinal vasculature upon genetic silencing or pharmacological inhibition of PFKFB3 in ECs (even though ±60% of glycolytic flux was retained) [18,19,30]. Conversely, overexpression of PFKFB3 resulted in increased glycolysis, subsequently promoting a pro-tip cell phenotype in mosaic endothelial spheroids, even in ECs with a strong genetic pro-stalk cell cue. On the other hand, while pro-angiogenic VEGF–VEGFR2 signalling enhances glycolytic flux in ECs via PFKFB3, blood flow-exerted laminar shear stress promotes Krüppel-like factor 2 (KLF-2) transcription factor to bind the PFKFB3 promoter, consequently repressing its transcription and maintaining a metabolically quiescent phenotype in mature vessels [31].

Alternatively, FGF signalling, through FGF receptors 1 and 3 (FGF-R1 and FGF-R3), has recently been shown to be a pivotal regulator of blood and lymphatic vascular development [32]. Indeed, recent findings have depicted a novel FGF-driven modulation of Myc, regulating hexokinase 2 (HK2) expression, a rate-limiting enzyme catalysing the first step of the glycolytic pathway. The FGF–Myc–HK2 axis was demonstrated to constitute a crucial driver of glycolysis in ECs with experiments using blood EC- and lymphatic EC-selective genetic deletion of FGF-R1 and FGF-R3, Myc or HK2 in mice, which suffered vascular defects [32].

While glycolysis has clearly been shown to support both the migratory tip cell phenotype and the proliferative stalk cell phenotype, the relative importance of glycolysis in tip versus stalk ECs has not been elucidated as yet. Future studies aiming to answer such questions could benefit from newer technologies including unbiased and untargeted multi-omics approaches and state-of-the-art transcriptomics such as single-cell RNA sequencing allowing the investigation of cell-to-cell variation.

### 3.2. Fatty acid oxidation and CPT1a

Besides the pivotal role of glycolysis, necessary for tip and stalk cell function during vessel sprouting, recent evidence unravelled the importance of the previously overlooked fatty acid oxidation pathway (FAO) during angiogenic processes [20]. Indeed, while tip cells have been demonstrated to rely primarily on a PFKFB3-driven glycolytic metabolism to rapidly produce enough ATP for vessel sprouting, stalk cells were also recently shown to depend on FAO, essential for vessel sprout elongation by sustaining the synthesis of deoxynucleotide triphosphates (dNTPs) [18,20]. FAO is a multistep metabolic pathway following the transport of FAs into the cell [33]. The addition of an acetyl-CoA moiety to these FAs permits their import into mitochondria via FAO's rate-limiting enzyme CPT1a. Once inside mitochondria, FAs undergo β-oxidation resulting in the production of acetyl-CoA, which further enters into the tricarboxylic acid (TCA) cycle. Entry of FA-derived acetyl-CoA, in conjunction with an anaplerotic substrate, sustained the TCA cycle for the production of aspartate, used for dNTPs synthesis and essential for DNA replication in proliferating ECs [20] (figure 1*b*). Interestingly, pharmacological blockade of CPT1 with etomoxir and EC-selective genetic deletion of CPT1a *in vitro* and *in vivo* resulted in a reduction of EC proliferation following a decrease in dNTPs synthesis, which in turn leads to impaired vessel sprouting [20]. Etomoxir also induced hyperpermeability *in vitro* and blood vessel leakage *in vivo* due to the alteration of calcium signalling [34].

Furthermore, FAO and CPT1a have been shown to modulate lymphangiogenesis. The mechanism via which the metabolism of lymphatic ECs (LECs) tunes lymphatic development has been elucidated only very recently [22]. Wong *et al*. documented the pivotal role of FAO and its key enzyme CPT1a in the lymphatic development of murine embryos; fully functional FAO in LECs was shown to promote lymphatic vessel growth and to stimulate LEC proliferation by sustaining the TCA cycle, in conjunction with an anaplerotic substrate, and promoting dNTP synthesis for DNA replication. In addition, FAO promotes the venous-to-lymphatic EC differentiation switch via epigenetic mechanisms. Indeed, Prox1, a LEC marker and central lymphatic transcription factor, enhances the transcription of the gene coding for CPT1a, which in turn increases FAO [35]. As a result, additional acetyl-CoA moieties are produced and used by the histone acetylase p300 for histone acetylation, preferentially at lymphangiogenic over angiogenic genes, resulting from an interaction of p300 with Prox1. This further promotes lymphatic gene expression and venous-to-lymphatic EC differentiation (figure 1*c*). As such, Prox1 is a crucial transcription factor able to activate lymphatic gene transcription epigenetically by manipulating FAO in LECs. This study illustrates how metabolism, in particular FAO, can regulate LEC differentiation and lymphatic vessel development via a combination of metabolite signalling and epigenetic regulation.

CPT1 inhibition in blood vessels was also reported to show potential therapeutic benefit by blocking pathological angiogenesis [20]. Indeed, the treatment of mouse pups with etomoxir reduced pathological ocular angiogenesis in a model of retinopathy of prematurity (ROP) through inhibition of EC proliferation. Interestingly, FAO and CPT1a also promoted dNTP synthesis in lymphatic vessels, as in vascular blood vessels, and CPT1 blockade by etomoxir reduced pathological injury-induced lymphangiogenesis [22]. Notably, the treatment of etomoxir-treated mice with acetate (which can be metabolized to acetyl-CoA) rescued the impaired lymphangiogenesis, raising the question whether acetate or other sources of acetyl-CoA might constitute an attractive therapeutic approach for the treatment of lymphoedema related to lymphatic vessel defects.

### 3.3. FOXO1: a gatekeeper of endothelial quiescence

**Figure 2.**
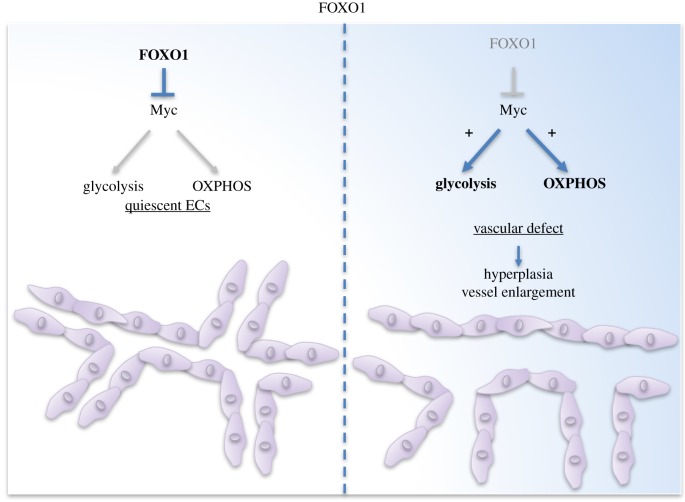
FOXO1 regulates vascular homeostasis. FOXO1 is a transcription factor ensuring EC quiescence and vascular homeostasis via c-Myc regulation. FOXO1 deletion in ECs causes severe vascular defect mediated by dysregulated EC metabolism controlled by c-Myc.

FOXO1 is part of the forkhead box O (FOXO) proteins, a sub-group of the FOX transcription factors family, composed of four members: FOXO1, FOXO3, FOXO4 and FOXO6. FOXO1 is ubiquitously expressed and plays a pivotal role in the regulation of genes implicated in cell growth, survival and differentiation [36]. This is crucial for embryo development as it was established that FOXO1 knock-out mice (*foxo1*^−/−^) display a strong vascular formation defect [37–39]. Mechanistically, FOXO1 constitutes a downstream effector of Akt and is necessary for vascular homeostasis [40]. Following growth factor activation, the PI3K/Akt pathway blocks FOXO1 activity through phosphorylation of three of its residues resulting in FOXO1's export from the nucleus to the cytoplasm [41]. Recent investigations highlighted the central role of FOXO1 as a key metabolic checkpoint and proliferation regulator in ECs [25]. FOXO1 acts as a gatekeeper of EC quiescence through inhibition of Myc, a key transcriptional factor in growth and anabolic metabolism, ultimately resulting in decreased glycolysis and impaired mitochondrial function (figure 2). Of note, embryonic lethality, hyperplasia and vessel enlargement were documented following endothelial FOXO1 gene deletion in mice. On the other hand, FOXO1 overexpression in EC causes defective vessel development and hypobranching, as such demonstrating the significance of FOXO1/Myc interplay in angiogenesis and vascular homeostasis [25]. In pulmonary artery remodelling, FOXO1 expression is decreased, which leads to Bad upregulation and Bcl-2 downregulation, two key proteins regulating programmed cell death. This interplay between FOXO1 and apoptosis in ECs may be a possible mechanism leading to pulmonary artery remodelling, as apoptotic ECs lose their ability to control smooth muscle proliferation [42]. Finally, FOXO1 promotes sprouting and migration of LECs, by enhancing the expression of the purigenic receptor P2RY1 in response to exogenous ATP [43]. These recent findings raise the question whether FOXO1 has a contextual function in the different EC subtypes.

### 3.4. Glutamine and asparagine metabolism in ECs

Glutamine is the most abundant non-essential amino acid circulating in human plasma (its physiological concentration in human plasma is 0.6 mM). Glutamine constitutes an important metabolic substrate as it: (i) contributes to sustaining cellular bioenergetics; (ii) supports the generation of biosynthetic precursors and macromolecules, and is used for protein synthesis; (iii) promotes nitrogen transfer for the generation of nucleotides, hexosamine and asparagine; and (iv) is one of the upstream regulators of mTORC1, known to promote protein synthesis and support cell growth [44,45]. Glutaminase, the first enzyme of glutamine catabolism, converts glutamine to glutamate and ammonia, while glutamate dehydrogenase metabolizes glutamate to α-ketoglutarate [46]. α-Ketoglutarate is used as carbon source for anaplerotic replenishment of the TCA cycle [47], while glutamate is a precursor of glutathione for the maintenance of cellular redox homeostasis [44]. Glutamate-derived nitrogen is also used for the synthesis of non-essential amino acids in transaminase reactions converting glutamate to α-ketoglutarate, while glutamate can be also converted to ornithine to generate polyamines and nitric oxide (NO), both pro-angiogenic factors [48,49].

**Figure 3.**
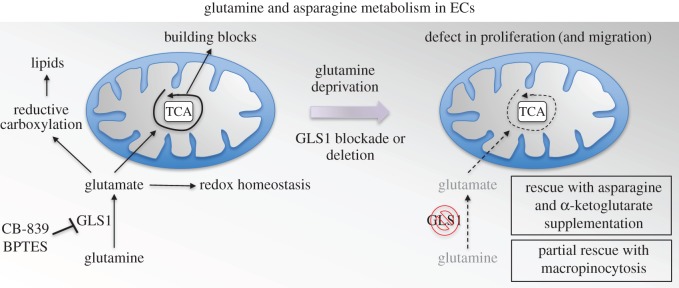
Glutamine and asparagine in angiogenesis. Glutamine plays a key role in EC metabolism: it constitutes a major precursor for macromolecules biosynthesis and the main substrate for TCA cycle anaplerosis, it participates in redox homeostasis and is also shown to be a precursor for lipid biosynthesis in ECs through reductive carboxylation. Glutamine deprivation or GLS1 blockade/deletion leads to a severe defect in proliferation and migration in ECs. Asparagine supplementation (together with α-ketoglutarate) rescues the phenotype showing an interlink between glutamine catabolism pathway and asparagine synthesis via ASNS. Proliferative defect following glutamine deprivation or GLS1 blockade/deletion in ECs can be partially rescued through macropinocytosis.

Over the past years, solid tumour and leukaemia cells have been documented to have a high avidity for glutamine (among other amino acids) in the field of cancer metabolism, yet little is known about glutamine metabolism and its implication in EC homeostasis and angiogenesis [50]. Only a few recent studies investigated the role of glutamine in EC proliferation, migration and sprouting [23,24]. Indeed, glutamine-deprived ECs, ECs with a selective genetic deletion of GLS1 or treated with a GLS1-specific inhibitor CB-839 showed impaired EC proliferation, migration and vessel sprouting *in vitro* and *in vivo* [23] (figure 3). Additional chimeric sprouting experiments using red mCherry^+^ control ECs and green GFP^+^ GLS1^KD^ ECs in mosaic spheroids emphasized the importance of glutamine metabolism and GLS1 for ECs to obtain the tip cell position during vessel sprouting [23].

Of interest, glutamine metabolism interlinks with asparagine metabolism in ECs [23]. Indeed, asparagine (together with an anaplerotic carbon donor such as α-ketoglutarate) was the only non-essential amino acid able to fully rescue the proliferative defect of glutamine-deprived ECs [23]. Little is known about the role of asparagine in EC biology. ECs can take up asparagine from the blood (when available), but they can also use ASNS to produce this amino acid de novo by fusing glutamine-derived nitrogen with aspartate, in nutrient-limited environments, such as occurs in the tumour milieu or when ECs migrate into avascular regions [23]. ECs may additionally rely on ASNS when its expression levels are upregulated (by hypoxia, starvation of glucose or amino acids, or endoplasmic reticulum (ER) stress) or when extracellular asparagine levels are limiting (upon protein limitation, unbalanced amino acid intake, asparaginase treatment, in certain cancers, etc.) [51–55].

Mechanistically, asparagine rescues the proliferation defects of glutamine-deprived ECs by increasing protein synthesis, lowering ER stress and reactivating mTOR signalling [23]. Interestingly, asparagine has long been thought to only function as a precursor of protein synthesis, in contrast to the other nineteen amino acids. However, increasing evidence suggests a possible role of this amino acid as a signalling metabolite that senses metabolic fuel reserves and availability, and coordinates cellular homeostatic responses accordingly [56]. Indeed, intracellular asparagine levels are the lowest of all non-essential amino acids in proliferating cells, and asparagine exclusively relies on glutamine as nitrogen donor, suggesting that asparagine is a cellular rheostat that senses the availability of TCA cycle intermediates and the supply of reduced nitrogen for biosynthesis of non-essential amino acids [56]. Thus, upon amino acid and carbohydrate starvation, ECs may upregulate asparagine synthesis, to warn the cell about insufficient substrate availability for continued cell division. Whether asparagine functions as a signalling metabolite in ECs is an outstanding question. Nonetheless, GLS1 and ASNS may constitute new attractive therapeutic targets for the development of novel anti-angiogenic strategies targeting EC metabolism.

Another study confirmed glutamine's role in EC metabolism and supported the findings that glutamine indeed is a main substrate for the TCA cycle, acts as a nitrogen donor and also showed that glutamine may participate in lipid metabolism through reductive carboxylation [24]. Inhibition of glutamine catabolism via BPTES or genetic deletion of GLS1 in ECs affected proliferation. This was attributed to a collapse of oxidative metabolism in ECs following a decrease in the levels of TCA intermediates (figure 3). Interestingly, glutamine-deprived ECs undergo macropinocytosis (endocytosis of nutrients and macromolecules from the extracellular environment) in order to fulfil their need for nitrogen [24]. This mechanism, however, could not rescue EC growth upon glutamine starvation, highlighting the key importance of glutamine as a vital fuel.

Of note, the role of glutamine as a carbon source to supply the TCA cycle in ECs was also demonstrated in PAH, both in a mouse model and in human patients [57]. Indeed, increased glutamine catabolism, (over)supplying the TCA cycle, was observed in pulmonary ECs of patients with a BMPR2 (bone morphogenetic receptor type II) mutation suffering chronic PAH (PAH classified as group I by WHO). This metabolic maladaptation supports EC survival and a hyperproliferative phenotype, presumably through enhanced energy and biomass production [57]. Mechanistically, oxidative stress-mediated reduction in sirtuin-3 (SIRT3) activity (previously implicated in PAH pathogenesis) increases HIF-1*α* activity, which could explain the augmentation in glutamine utilization by pulmonary microvascular ECs from BMPR2-mutant PAH mice [57]. In such pathological circumstances, targeting glutamine catabolism may represent an appealing therapeutic strategy that could benefit PAH patients.

## 4. Therapeutic implications

The success of anti-VEGF therapies in the clinical setting is limited by insufficient efficacy, intrinsic refractoriness and acquired resistance [58]. Hence, novel therapeutic approaches based on the modulation of newly described actors of EC metabolism are emerging as an appealing alternative to treat pathological angiogenesis. As scientific efforts recently deciphered the pivotal role of EC metabolism in angiogenesis, key metabolic enzymes came into the spotlight as possible therapeutic targets to modulate pathological vascular outgrowth, namely PFKFB3, CPT1a, GLS1 and ASNS. In this section, we briefly discuss the main small molecule compounds developed so far, as well as their future clinical development as new anti-metabolic agents (table 1).

**Table 1.** Main inhibitors of metabolic enzymes involved in EC metabolism and angiogenesis. List of compounds, target information and current status in the (pre)clinical pipeline of major inhibitors respectively blocking PFKFB3, CPT1, GLS1 and ASNS.

target	drug or compound	current status
PFKFB3	3PO	preclinical
	PFK-158	clinical Phase I
	phenoxyindole	preclinical
CPT1	etomoxir	preclinical
	perhexiline	preclinical
GLS1	BPTES	preclinical
	compound 968	preclinical
	CB-839	clinical Phase II
ASNS	*N*-acylsulfonamide 6	preclinical
	adenylated sulfoximine	preclinical

PFKFB3-driven glycolysis is essential for angiogenesis, as it provides ATP to sustain the angiogenic switch. Thus, blocking PFKFB3's activity in the context of a pathological angiogenesis constitutes an attractive therapeutic strategy. Small inhibitor and tool compound 3-(3-pyridinyl)-1-(4-pyridinyl)-2-propen-1-one (3PO) was demonstrated to lower glycolysis in pathological tumour angiogenesis [19]. 3PO only partially and transiently lowers EC glycolysis [19], as such avoiding deleterious systemic effects on glycolysis-dependent healthy tissues. Moreover, treatment with a low dose of 3PO (i.e. lower than the 3PO concentrations used for treating cancer cells [59]) was sufficient to reduce metastatic spread *in vivo* in several tumour-bearing mouse models, as a result of tumour vessel normalization [21]. 3PO-induced tumour vessel normalization also improves the efficacy of chemotherapy, in part, by ameliorating drug delivery. Importantly, as (tumour) ECs are so glycolysis-addicted, even a reduction of glycolysis in these cells by 15–20% suffices to induce tumour vessel normalization [21]. In fact, the use of a high dose of 3PO or the use of 2-deoxyglucose (which both lower glycolysis by greater than 70–90%) is toxic for ECs *in vitro*, and no longer evokes vessel normalization, but instead incites vessel disintegration, thereby facilitating cancer cell escape and dissemination [60]. Overall, whether the ‘less is more’ paradigm shift is also relevant in the human cancer setting requires further study, but these preclinical studies nonetheless raise concerns about the unconsidered use of ‘maximal tolerable doses' of PFKFB3 blockers.

Meanwhile, a new class of 3PO derivatives has been synthesized in an attempt to improve the pharmacokinetic properties and toxicological parameters of the drug candidate in a study focusing on the inhibition of PFKFB3-driven glycolysis in cancer cells. Among this new class, 1-(4-pyridinyl)-3-(2-quinolinyl)-2-propen-1-one (PFK-158) demonstrated the therapeutically most desirable parameters and recently entered Phase I clinical trials, in a dose-escalation study in patients with advanced solid malignancies (clinicaltrials.gov) [61]. Finally, a recent structure-guided synthesis and screening of a chemical collection led to the discovery of a new PFKFB3 inhibitor compound, a phenoxyindole derivative, exerting a higher selectivity for PFKFB3 over the other PFKFB isoforms [62]. Its direct interaction with PFKFB3's ATP-binding pocket was elucidated using the crystallographic structure of PFKFB3's catalytic domain.

Recent investigations also showed the beneficial therapeutic effect of CPT1 inhibition by etomoxir, as it reduced EC proliferation and pathological angiogenesis in mouse pups with ocular retinopathy (ROP model) *in vivo* [20]. Etomoxir, a small 2-oxiranecarboxylate derivative, is an irreversible inhibitor of CPT1 [63]. Interestingly, etomoxir gained interest from the cancer metabolism field as a new therapeutic approach as it reduced the tumour growth of several FAO-dependent tumour models [64]. Perhexiline, originally developed as an anti-anginal agent, is another inhibitor of CPT1 (and CPT2), but its therapeutic use is limited [65]. Additional CPT1 inhibitors with improved pharmacological profile are thus mandated.

Recent years of investigation in tumour metabolism highlighted the reliance of solid tumour and leukaemia cells on glutamine, which has fostered the development of GLS1 inhibitors. The application of these inhibitors might be repurposed for the treatment of pathological vascular outgrowth associated with ocular diseases and cancer, as recent insights into EC metabolism have depicted a pivotal role of GLS1 in angiogenesis [23,24]. BPTES is an irreversible allosteric inhibitor targeting glutaminase, but exhibits moderate efficacy as GLS1 inhibitor, poor stability and a limited solubility [66]. Compound 968 is another non-competitive allosteric inhibitor of GLS1 with anti-proliferative effects on glutamine-addicted cancer cells, but its hydrophobic properties limit its therapeutic use for *in vivo* studies and further preclinical development [67]. CB-839 is a potent, selective and reversible inhibitor of human GLS1 with favourable oral bioavailability properties [68]. Preclinical studies demonstrated the effect of CB-839 on the progression of various tumour models [68,69]. Cancer patients are enrolled for a Phase Ib clinical study aiming to combine CB-839 with standard chemotherapeutic agents and immuno-oncology compounds. In addition, a Phase II clinical trial recruiting patients with renal carcinoma and triple negative breast cancer has commenced in 2017 (calithera.com and clinicaltrials.gov).

Finally, the novel metabolic interlink between glutamine and asparagine for the regulation and support of EC metabolism opens new horizons for drug development of new molecules targeting asparagine metabolism through inhibition of ASNS [23]. Only a very few studies using ASNS inhibitors have been published so far, raising hope that the recent findings on the role of ASNS in angiogenesis will further encourage its drug discovery. *N*-acylsulfonamide compound 6 inhibits human ASNS and has been proposed as an alternative treatment of drug-resistant leukaemia [70]. The adenylated sulfoximine also blocks human ASNS with an improved affinity (nanomolar range) and was employed as a possible treatment for asparaginase-resistant leukaemia cells [71]. More recently, efforts have been made in order to synthesize new sulfoximine derivatives with improved metabolic stability and bioavailability [72,73]. Another possible route, worthwhile to be considered, is the combined treatment of an ASNS blocker together with asparaginase treatment, in order to not only deplete extracellular asparagine (which would reactively upregulate ASNS expression) but also block de novo synthesis of asparagine.

## 5. Conclusion and perspectives

EC metabolism has emerged as a novel key regulator of vascular development and pathological angiogenesis. These findings pave a novel, exciting but challenging road for the development of anti-angiogenic strategies based on targeting EC metabolism targets. To date, the function of only a few key metabolic enzymes in ECs has been characterized; however, these findings have shed light on the pivotal role of metabolism in vascular biology, constituting a basis to foster the development of new anti-metabolic drugs. The realization that metabolism comprises thousands of drug-able enzymes and transporters offers unprecedented opportunities to develop novel anti-angiogenic agents.

State-of-the-art technologies, including metabolomics profiling and single-cell transcriptomics, promise to yield answers to more intricate outstanding questions. Further, new opportunities lie in the study of, for example, the metabolic difference between tip, stalk and phalanx ECs. However, they can also reach beyond ECs alone, such as the investigation of metabolic crosstalk between ECs and their surrounding cell types (stromal cells, immune cells and cancer cells). In addition, how such metabolic crosstalk influences signalling and the functional outcome of ECs and their neighbouring cell types is another interesting yet outstanding question. This may especially be interesting in the study of EC dysfunction in diseases such as cancer, diabetes, atherosclerosis, ocular pathologies, etc. Furthermore, untargeted and unbiased ‘(multi-)omics’ approaches (metabolomics, proteomics, transcriptomics, epigenomics, etc.) in combination with *in silico* genome-scale metabolic modelling (GEM) will enable a data-driven forward (phenotype-to-genotype) approach that may uncover novel metabolic targets in both health and diseased states, ultimately yielding exciting insights for future therapeutic avenues.

## Data accessibility

This article has no additional data.
